# Detection and characterization of putative hypervirulent *Klebsiella pneumoniae* isolates in microbiological diagnostics

**DOI:** 10.1038/s41598-023-46221-w

**Published:** 2023-11-03

**Authors:** Bernd Neumann, Claudia Stürhof, Anca Rath, Bärbel Kieninger, Elias Eger, Justus U. Müller, Alexander von Poblocki, Nadja Gerlitz, Paul Wollschläger, Wulf Schneider-Brachert, Katharina Schaufler, Kathleen Klaper, Jörg Steinmann

**Affiliations:** 1 Institute of Clinical Microbiology, Infectious Diseases and Infection Control, Paracelsus Medical University, Nuremberg General Hospital, Nuremberg, Germany; 2 Department of Infection Prevention and Infectious Diseases, University Hospital Regensburg, Regensburg, Germany; 3 Department of Epidemiology and Ecology of Antimicrobial Resistance, Helmholtz Institute for One Health, Helmholtz Centre for Infection Research HZI, Greifswald, Germany; 4 Pharmaceutical Microbiology, Institute of Pharmacy, University of Greifswald, Greifswald, Germany; 5 Division 18–Sexually transmitted bacterial Pathogens and HIV, Department of Infectious Diseases, Robert Koch Institute, Berlin, Germany; 6 Institute of Hospital Hygiene, Medical Microbiology and Infectious Diseases, Paracelsus Medical University, Nuremberg General Hospital, Prof.-Ernst-Nathan-Str. 1, 90419 Nuremberg, Germany

**Keywords:** Clinical microbiology, Bacterial infection

## Abstract

Hypervirulent *Klebsiella pneumoniae* strains (hvKp) can cause invasive community-acquired infections in healthy patients of all ages. In this study, the prevalence of putative hvKp in a German tertiary center was investigated and hvKp were characterized by phenotypic and molecular assays. All *K. pneumoniae* isolates in routine microbiological diagnostics from a single center were screened by string-testing over a period of 6 months. String-test positive (≥ 0.5 mm) isolates were re-evaluated on different media and under various conditions (aerobe, anaerobe). For string-test positive isolates, genes (magA, iutA, rmpA and rmpA2) associated with hypermucoviscosity and hypervirulence were amplified by multiplex PCR. PCR-positive isolates were subjected to whole-genome sequencing and sedimentation and biofilm formation assays. From 1310 screened *K. pneumoniae* isolates in clinical routine 100 isolates (7.6%) were string test positive. From these, 9% (n = 9) were defined as putative hvKp (string-test+/PCR+). Highest rate of string-test-positive isolates was observed on MacConkey agar under aerobic conditions. Amongst these nine putative hvKp isolates, the international lineage ST23 carrying hvKp-plasmid pKpVP-1 was the most common, but also a rare ST86 with pKpVP-2 was identified. All nine isolates showed hypermucoviscosity and weak biofilm formation. In conclusion, 9% of string-positive, respectively 0.69% of all *K. pneumoniae* isolates from routine were defined as putative hypervirulent. MacConkey agar was the best medium for hvKp screening.

## Introduction

*Klebsiella pneumoniae* (*K. pneumoniae*) is an opportunistic Gram-negative pathogen emerging worldwide^1,2^. The international spread is driven by different clonal genetic lineages as well as carriage of various plasmids, unclosing selection advantages such as antimicrobial resistances^1^. Various infections are caused by *K. pneumoniae*, including sepsis and pneumonia^3,4^. These “classic”, usually antimicrobial-resistant *K. pneumoniae* (cKp) typically affect multimorbid or immunocompromised patients in healthcare facilities^5,6^.

Beside this incumbent position among other hospital pathogens, *K. pneumoniae* stands out since the first descriptions of hypervirulent *K. pneumoniae* (hvKp) variants in Asia^7–9^. Nowadays, these hvKp pathotypes are reported on an international scale^10–13^. HvKp are characterized by a unique clinical manifestation, usually resulting in pyogenic liver abscesses and metastatic spread to multiple infection sites^14^. Primarily, hvKp affect healthy, young and immunocompetent persons that acquire the infection outside the hospital and healthcare environment^14^.

Hypervirulence seemed to be correlated with a hypermucoviscous (hmv) phenotype, but this association is flawed and still under discussion^15–17^. The hmv phenotype seems to rely on *rmpA*/*rmpA2*-associated enhanced production of polysaccharides and can regularly be identified by so called string-test^16,18–20^.

Recently, genome sequencing revealed potential molecular hvKp marker genes (*rmpA*, *rmpA2*, *iutA*, *peg-344*, *iroB*), almost-exclusively located on hypervirulence-mediating plasmids, as well as capsule gene *magA* associated with K1 serotype^14,21–26^. The dissemination of hvKp clonal lineages, carrying these plasmids, facilitate the spread of hypervirulence in cKp populations; several multidrug-resistant hvKp were reported for the first time, including so called hybrid plasmids coding for both hypervirulence and resistance traits^26–28^.

In Germany, lately individual case reports and hospital outbreaks of hvKp were also reported^23,29–31^. Information about general dissemination and epidemiology of hvKp is still fragmentary for Germany. Since there is no routine screening or a systematic surveillance established for hvKp, our study intended to implement a multi-step test panel to identify and characterize putative hvKp isolates in a routine diagnostic laboratory in a tertiary care hospital (> 2200 beds) in Germany. This panel was assessed to identify an early detection method for hvKp directly from agar plates in routine diagnostics. Putative hvKp were characterized by whole-genome sequencing, a sedimentation assays and biofilm formation capability.

## Material and methods

### Sampling of bacterial isolates

All clinical isolates of *K. pneumoniae*, obtained of differing patient material origin such as blood, urine, etc. were initially string-tested in the microbiological routine laboratory. This initial string-test was realized in primary diagnostics including all specimen-cultivations on different solid media such as Mueller–Hinton, Columbia blood and MacConkey agar. The laboratory received the specimen from five hospitals. Isolates were collected over 6 months in 2021. All colonies initially string-tested positive were subjected to species identification by matrixed-assisted laser desorption ionisation time-of-flight (MALDI-TOF) mass spectrometry (Bruker Daltonik GmbH, Bremen, Germany).

### String-testing on different media

Initially string-test-positive isolates were cultivated on three different solitude media that are commercially available and commonly used in routine diagnostics: Mueller–Hinton (MH; Xebios Diagnostics, Düsseldorf, Germany), Columbia blood agar (Xebios Diagnostics, Düsseldorf, Germany) and MacConkey agar (Mast Diagnostica, Reinfeld, Germany) plates. The incubation was realized at 36 ± 1 °C; 24 h under aerobic condition or 48 h under anaerobic (0.15% O_2_, 9.9% CO_2_, 9.9% H_2_, 80.05% N_2_) conditions using a jar gassing system (Meintrup DWS, Herzlake, Germany). Each isolate was cultivated under these six different conditions. The string-test was realized for all isolates and growth conditions. The verification as positive was a mucoid string of ≥ 5 mm, when touching bacterial plate growth with a standard inoculation needle and gentle pulling away, as described before^20^. All mucoid strings were measured to maximum length using a scale with millimetre markings.

### PCR screening for virulence genes

The initially string-test-positive isolates were subjected to a modified multiplex-PCR based on Compain et al*.*^32^. For DNA extraction, the semi-automated benchtop device GenoXtract (Hain Lifescience GmbH, Nehren, Germany) was used, according to the manufacturer’s instructions. The PCR screening included four gene targets *rmpA*, *rmpA2*, *iutA* and *magA* (Table 1). The multiplex-PCR was realized as 25 µL formulation, using 2 µL extracted DNS, 12.5 µL DreamTaq Master Mix (Thermo Fisher Scientific, Waltham, MA, USA), 0.5 µL nuclease-free water and primer solution as stated in Table 1. As positive control, the *K. pneumoniae* isolate 316/15 was used^33^. The PCR conditions were realized as described by Compain et al.^32^.

**Table 1:** Primers used for the multiplex-PCR.

Primer	Used volume	Sequence	Product size (bp)	Reference GenBank
*magA*-f	0.5 µL (4 µM)	GGTGCTCTTTACATCATTGC	1283	AY762939
*magA*-r	0.5 µL (4 µM)	GCAATGGCCATTTGCGTTAG	1283	AY762939
*rmpA*-f	1.5 µL (10 µM)	ACTGGGCTACCTCTGCTTCA	535	KY403906.1
*rmpA*-r	1.5 µL (10 µM)	CTTGCATGAGCCATCTTTCA	535	KY403906.1
*rmpA2*-f	2.0 µL (2 µM)	TTATGTGCAATAAGGATGTT	620	NC_005249.1
*rmpA2*-r	2.0 µL (2 µM)	CTAGGTATTTGATGTGCAC	620	NC_005249.1
*iutA*-f	1.0 µL (1 µM)	GGGAAAGGCTTCTCTGCCAT	920	AY378100
*iutA*-r	1.0 µL (1 µM)	TTATTCGCCACCACGCTCTT	920	AY378100

### Hypermucoviscosity sedimentation assay

The hmv phenotype of putative hvKp isolates was quantified as the OD_600_ ratio using the hypermucoviscosity sedimentation assay as described by Eger et al.^31^. Each PCR-positive isolate was cultured in LB broth at 37 °C and 130 rpm for 24 h. Then, 1.5 mL of the bacterial suspension was centrifuged (1000×*g*, 5 min, room temperature), resulting supernatants were transferred to 96-well microtiter plates, and OD_600_ was measured. The OD_600_ of the cultures was also measured and the ratio of supernatant to culture OD_600_ was calculated. The assay was performed in triplicates and using a well-characterized hvKp ST420 (PBIO2030) as positive control and ATCC700603 as a non-hmv control^31^.

### Biofilm formation

The clinically important ability to form biofilms was tested for the putative hvKp isolates using a crystal violet assay as described previously^34^. Briefly, overnight cultures were diluted 100-fold in 5 mL of fresh M9 minimal salt medium (MP Biomedicals, Irvine, CA, USA) containing 0.4% (w/v) glucose (Carl Roth, Karlsruhe, Germany) and incubated at 37 °C and 130 rpm until OD600 reached 0.5 McFarland standard turbidity. The suspensions were diluted 1:100 and transferred in triplicate to 96-well microtiter plates. After incubation for 24 h at 28 °C, OD_600_ was measured using sterile medium as blank. Planktonic cells were then removed and adherent biofilms were washed thrice with deionized water and dried for 10 min. Dried biofilms were fixed using 250 µL of methanol (99% *v/v*) for 15 min and again dried at room temperature. The biofilms were stained using 250 µL of 0.1% (*w/v*) crystal violet solution for 30 min. After staining, unbound dye was removed by washing three times with deionized water and dried for 10 min. Subsequently, bound crystal violet was dissolved at room temperature using 300 µL of a mixture of 80 parts ethanol (99.8% (*v/v*); Carl Roth, Karlsruhe, Germany) and 20 parts acetone (Merck, Darmstadt, Germany) with horizontal shaking at 200 rpm for 30 min. A total of 125 µL was transferred to a new 96-well microtiter plate and measured at OD_570nm_. The specific biofilm formation (SBF) was calculated using the formula by Niu et al.^35^: SBF = (B − NC)/G, where B is the OD_570_ of stained biofilm, NC is the OD_570_ of stained control wells, and G is the OD_600_ representing the density of planktonically grown cells. The assay was performed in triplicates and using the strains W3110 as a strong biofilm-forming control and PBIO729 as a weak biofilm-forming control^34^.

### Whole-genome sequencing

All PCR-positive isolates were subjected to whole-genome sequencing (WGS). The DNA for WGS extracted using the QIAmp DNA Mini Kit (#54304; QIAGEn Diagnostics GmbH, Hilden, Germany). The isolated DNA was quantified using a Qubit 4 fluorometer and the dsDNA HS Assay Kit (Thermo Fisher Scientific, Bremen, Germany). Illumina Nextera reagents (Illumina GmbH, Berlin, Germany) were used for library preparation.WGS was performed at Illumina NextSeq (Illumina GmbH, Berlin, Germany) platform and 2 × 150 nucleotide paired-end reads were obtained.

### Sequence data processing and downstream analyses

A de novo assembly was carried out with the paired-end reads as input, for which the assembler SKESA (v2.4.0) integrated in SeqSphere + (client v9.0.1, server v9.0.0,) was used^36,37^. The parameters Percentage Good cgMLST Targets (> 95.0), Average Coverage (Assembled) (> 100), Approximated Genome Size (Mbases) (5.2 to 5.8), and GC-Content (Assembled) (56.8 to 57.5), which was also integrated in SeqSphere+, were used as quality criteria for sequencing.

The de novo reconstructed sequences were used for strain characterisation. The species verification was realized using the Type Strain Genome Server (TYGS) online platform^38^. For insights in antibiotics resistance genes and mobile genetic element content, the web tools provided by the center for genomic epidemiology, including ResFinder (v4.1), PlasmidFinder (v2.1) and the MobileElementFinder (v1.0) were applied^39–41^. For screening of hypervirulence-associated genes, Kleborate was used^42^. The online platform Pathogenwatch was applied for MLST, cgMLST and cSNP-phylogenetic analyses^43^. All German *K. pneumoniae* isolates submitted to the Pathogenwatch database (n = 940, as of 1th August 2023) were used for a phylogenetic assessment of the study isolates. Visualization of phylogenies was realized using iTOL (v6.8)^44^.

### Statistical analyses

The statistical analysis of the string-length data was performed with the statistical program R (version 3.6.3). Normal distribution was tested and a mean comparison of the six independent data sets "aerobic—Columbia blood agar", "aerobic—MacConkey agar", "aerobic—Mueller–Hinton agar", "anaerobic—Columbia blood agar", "anaerobic—MacConkey agar", and "anaerobic—Mueller–Hinton agar" were performed. Significance was pairwise tested, applying the Dunn test (p < 0.05).

Further, investigation on string-lengths of PCR-positive *K. pneumoniae* strains compared to PCR-negative strains was realized. In each case, a mean comparison of the string-lengths of the two independent samples "PCR-negative" and "PCR-positive" was performed for the six cultivation conditions.

## Results

### Isolate collection

During 6 months of screening, 1310 clinical isolates of *K. pneumoniae*, obtained from 840 patients of differing material origin were string-tested in the microbiological routine laboratory. The 100 (7.63%) initially string-test-positive (≥ 5 mm string-length) isolates were collected and subjected to further analyses. Most isolates originated from urine (n = 58), followed by respiratory materials (n = 18), blood culture (n = 11) and various swab and drainage origin (n = 13). The 100 isolates were collected from 68 patients, of which 35 (51.47%) were female. Patients age ranged from 26 to 95 years [mean = 71; median = 73].

### String-test investigations (positivity and length)

To evaluate the influence of medium and condition to the string length the 100 isolates were subjected to string-testing on three standard agar media under two different conditions (aerobic/anaerobic). The string-test results for all isolates and conditions were summarized in Supplemental Data S1. The outcome of the string-test varied depending on the used culture medium and growth condition.

**Figure 1:**
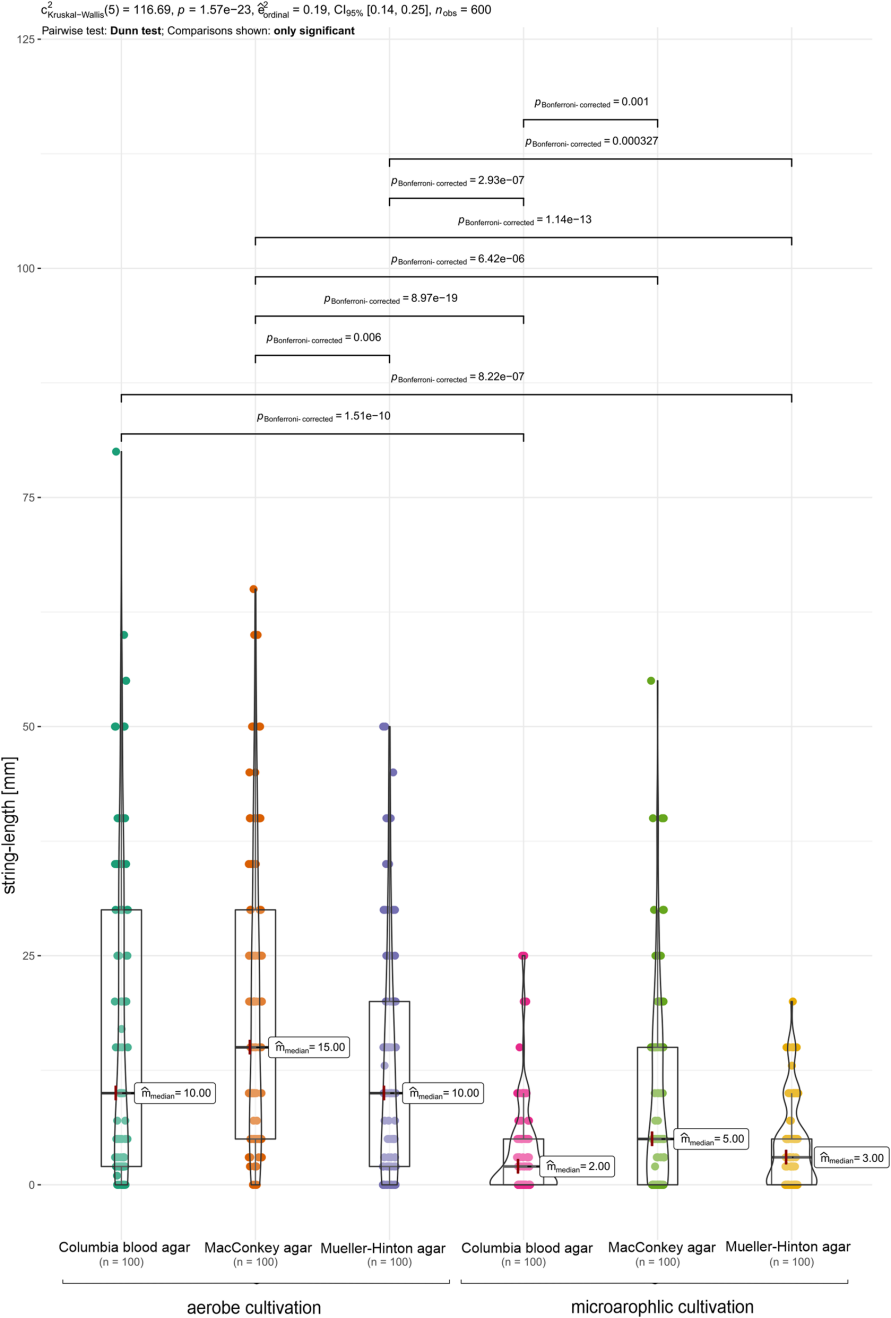
Comparison of observed string-lengths by cultivation method and cultivation condition (aerobe/anaerobe). The coloured dots represent one measured value per isolate. The values were depicted as violin box-plots. All significant differences (pairwise Dunn-test, p < 0.05) between conditions were highlighted above.

The highest rate of string-test-positive isolates was observed on MacConkey agar, followed by Mueller–Hinton agar. This observation was independent of cultivation condition, but the oxygen concentration had influence on the amount of string-test-positive isolates. Under anaerobic conditions, less isolates were assessed as positive. Regarding the measured string-lengths, also several differences could be observed (Fig. 1).

The measured string-lengths of all isolates were depicted in Fig. 1 and compared by agar media and cultivation condition. As shown, the string-lengths varied between the agar media with MacConkey agar resulting significantly the longest strings (aerobe media: 15 mm; anaerobe median: 5 mm). In general, the isolates generated significantly (p < 0.05) shorter strings under anaerobe conditions, comparing the different cultivation media (Fig. 1).

### Putative hypervirulence and string-test

The 100 isolates were further subjected to PCR screening for genes knows to contribute to hypermucoviscosity and hypervirulence potential in *K. pneumoniae*. Through multiplex PCR, nine isolates were identified as positive for the associated genes (Table 2). In Table 2 the isolates were listed and complemented with isolation material information.

**Table 2:** Information of PCR-positive isolates.

Isolate	*magA*	*iutA*	*rmpA2*	*rmpA*	Material	Patient sex	Patient age [years]
SPK008	+	+	+	+	Tracheal secretion	Male	72
SPK0017	+	+	−	+	Throat swab	Male	36
SPK0025	+	+	+	+	Blood	Male	61
SPK0027	+	+	+	+	Wound	Female	73
SPK0043	−	+	−	+	BAL	Male	73
SPK0052^a^	+	+	+	+	urine	Female	81
SPK0064	−	−	−	+	BAL	Male	57
SPK0077	+	+	+	+	Blood	Female	89
SPK0094^a^	+	+	+	+	Blood	Female	81

^a^Isolated of one patient with 2 months interval; BAL = bronchoalveolar lavage.

The virulence gene content of the isolates varied. In all isolates, *rmpA* and *iutA* were detected, except for isolate SPK0064 solely carrying *rmpA*. Six isolates additionally carried the gene *rmpA2*. Seven isolates showed a band for *magA*, which contributes to capsule formation of serotype 1.

The background of the positives was diverse, most from respiratory material (n = 4) and blood culture (n = 3). These isolates were obtained from five male and three female patients, with age ranging from 36 to 89 years. The strains SKP0052 and SPK0094 were isolated from urine and blood culture respectively, from the same patient at a 2 months interval.

**Figure 2:**
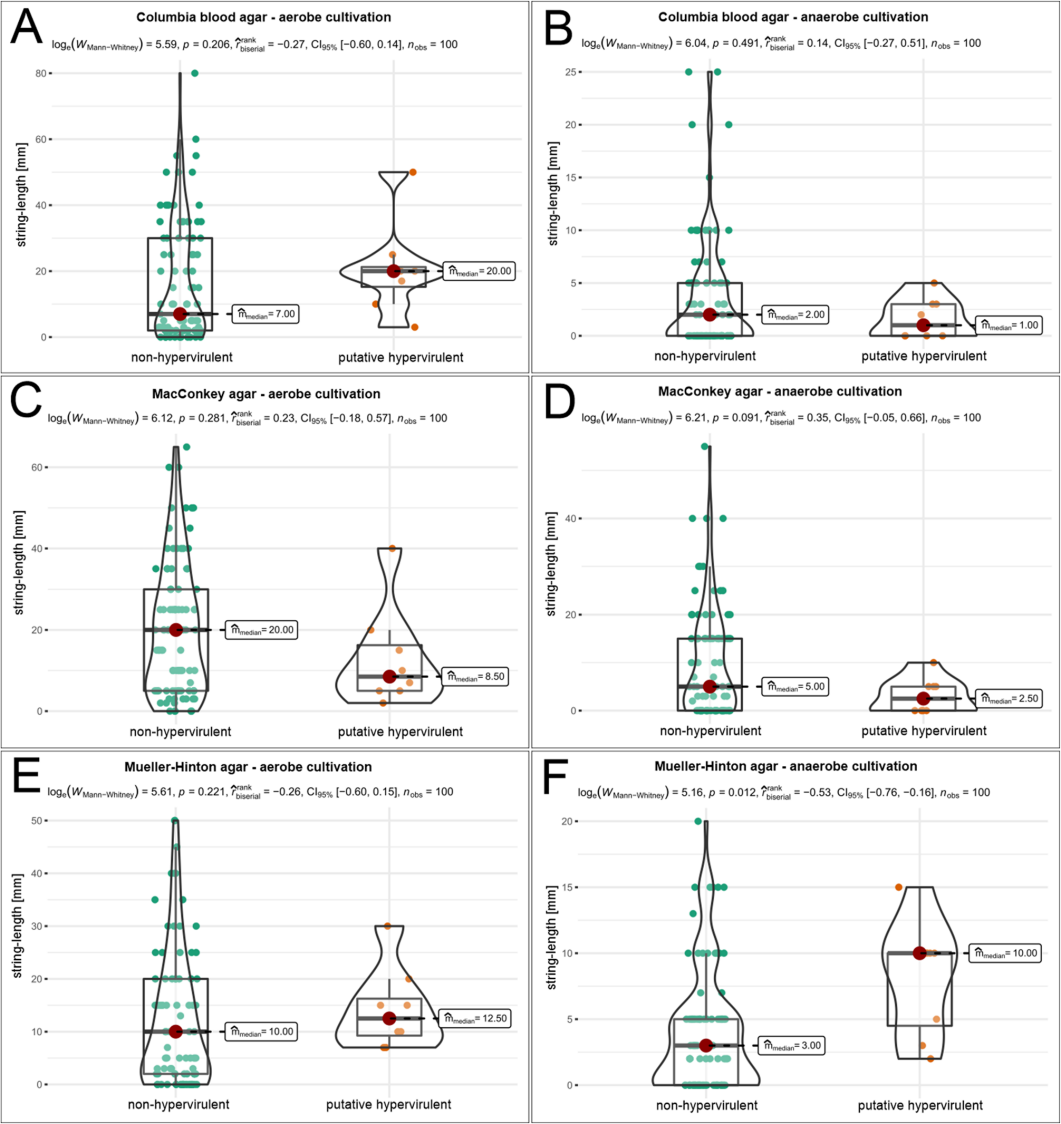
Comparison of median string-lengths of isolates carrying or not-carrying virulence genes identified by PCR. All cultivation parameters (**A**–**F**) were tested using the Mann–Whitney-U-Test for significance. The values were depicted as violin box-plots.

Further, the possible association of virulence gene and results in the string-testing was investigated. For this, the string-lengths of all realized conditions were compared for the two groups ‘non-hypervirulent’ and ‘putative hypervirulent’ (Fig. 2).

Several differences were observable between both aerobe and anaerobe cultivation as well as for each tested solid medium (Fig. 2A–F). The Mann–Whitney-U-Test revealed only under condition Mueller–Hinton anaerobe (Fig. 2F), a significant (p < 0.05) difference in string-length medians. The median string-lengths of isolates without virulence gene content was 3 mm, while PCR-positive tested isolates showed median string-length of 10 mm.

In general, the isolates carrying virulence genes showed high median strings lengths under aerobic cultivation, especially compared to the string-test threshold usually stated in literature, but not significant.

### Genomic and phylogenetic characterization

Based on string-test and PCR results, isolates assumed as putative hvKp were subjected to whole-genome sequencing for genomic characterization. In none of these isolates were acquired resistance genes for carbapenems identified.

**Figure 3:**
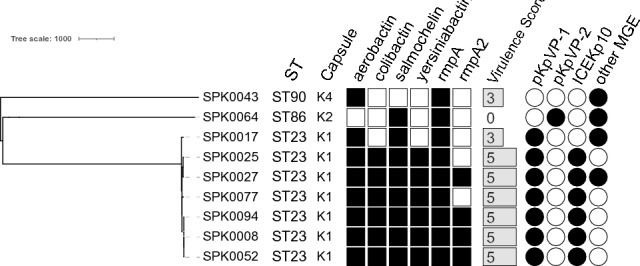
Results of genomic typing approaches and phylogenetic relations. Results based on analyses with Pathogenwatch including cgMLST-based phylogeny, MLST sequence types (ST) and the Kleborate tool for capsule typing and virulence factor identification (detectable: black square; undetectable: empty square). Based on these genes the virulence score (score range: 0-to-5; visualized as grey bars) was determined. Further, identified mobile genetic element (MGE) and plasmid content was visualized.

Based on cgMLST analyses, a phylogenetic tree of the nine isolates was calculated and typing results were visualized (Fig. 3).

As shown in Fig. 3, the nine isolates harboured diverse hypervirulence-associated genes. The WGS confirmed the results of the precedent multiplex PCR (see Table 2; *magA* associated with capsule type K1), except for *rmpA2* (SPK0025 and SPK0077). Sequencing data extended the typing by sequence type (ST), capsule type (K), colibactin, salmochelin, yersiniabactin and mobile genetic elements (MGE) content.

Seven isolates belonged to ST23 and capsule type K1. Of these, six were determined by Kleborate with virulence score of ‘5’. These isolates were also carrying the ICE*Kp10*, as well as the hypervirulence plasmid pKpVP-1. Isolate SPK0017 also carried pKpVP-1 and was virulence scored with ‘3’, based on missing the ICE*Kp10* that harbours colibactin and yersiniabactin. The strains SKP0052 and SPK0094, isolated from the same patient, were closely related but not identical since SPK0052 clustered closer together with SPK0008. The isolates SPK0043 and SPK0064 were not closely related with the other isolates of the screening collection, belonging to ST90/K4 and ST86/K2 respectively. These two isolates carried only two hypervirulence-associated genes, resulting in virulence scores of ‘3’ and zero (SPK0043). SPK0064 seem to carry the hypervirulence plasmid pKpVP-2. In SPK0043 the replicon ‘*repB*_KLEB’ was detected by the Kleborate tool, hinting a, possibly incomplete and chromosomal integrated, variant of the pKpVP-1 plasmid. Further, for SPK0043 was the carriage of other MGE predicted, including ColRNAI, IncFII(pSE11), IncR, repB(R1701), IncFIA(HI1) and pSM22. Also for SPK0027 were replicons for IncN and IncFII(pEHO1) plasmids detected, as well as IncHI1B(pNDM-MAR) for SPK0017 and SPK0064.

Further, the nine putative hypervirulent isolates detected through string-test screening were assessed regarding cgMLST-based phylogenetic relationships with other isolates from Germany (n = 940) submitted to the Pathogenwatch database (Supplementary Fig. S1). The isolates SKP0043 did not cluster together with other isolates. SPK0064 was also not part of a bigger cluster, but was related to another ST86 human isolate from blood, submitted in 2020. The other seven isolates clustered together with further six ST23 isolates. Of these, there was one isolates from milk powder, collected 2007 and three human isolates originating from blood, submitted 2020. Another was the human isolate 316/15, which was also used as PC in the multiplex-PCR and for the last isolate was only collection year 2018 as information available.

### Phenotypic characterization of putative hypervirulent isolates

**Figure 4:**
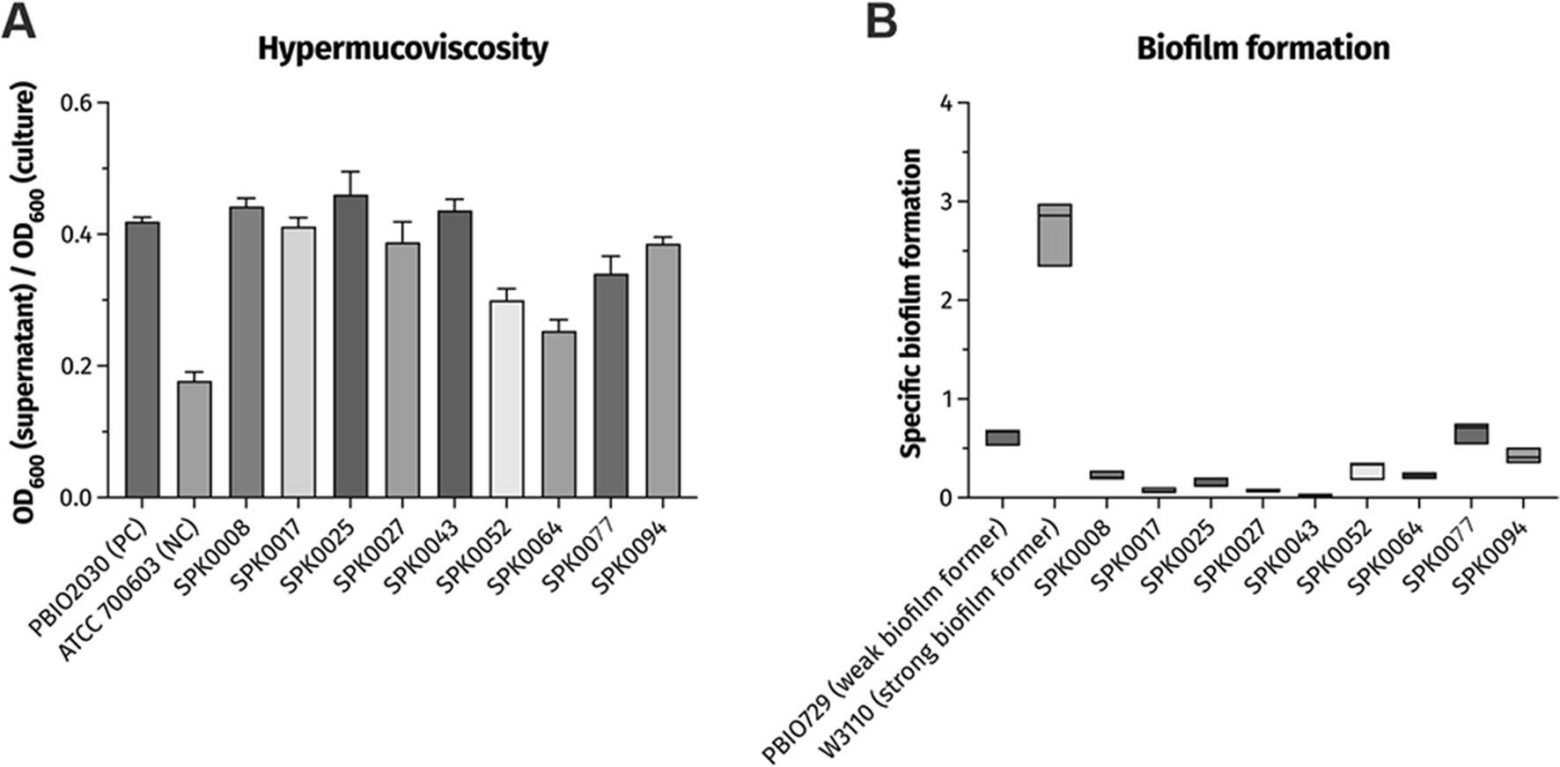
Results of phenotypic characterizations of PCR-positive *K. pneumoniae* isolates. (**A**) Quantification of mucoid phenotype by hypermucoviscosity sedimentation assay. Strain PBIO2023 was included as internal hvKp reference, and ATCC700603 served as a non-hmv control. (**B**) Results of the crystal violet-based biofilm assay, visualized as specific biofilm formation (SBF). The strains PBIO729 and W3110 were used as internal controls for a weak and strong biofilm formation, respectively.

The nine isolates with positive results in the multiplex-PCR were subjected to phenotypic characterization focussing on the hmv phenotype, a key characteristic of hvKp isolates. Results of the hypermucoviscosity sedimentation assay were visualized in Fig. 4A.

As depicted in the bar chart, all isolates showed increased mucoid phenotypes compared to the negative control (NC). The hmv ratios varied comparing the isolates, whereas SPK0008, SPK0025 and SPK0043 showed more sedimentation than the PC, the hmv phenotypes was less distinctive in SPK0052 and SPK0064. The latter was the only isolate in the study with a virulence score of 0, solely carrying *rmpA* and *iro* as virulence genes; while SPK0052 showed a virulence score of 5, tested positive for all gene targets. Interestingly, SPK0052 and SPK0094 showed varying results in this assay, albeit isolated from the same patient and almost identical on genome level.

Further, the biofilm formation, a generally important characteristic for pathogenicity and resistance against host factors, was phenotypically investigated for the putative hypervirulent isolates (Fig. 4B). This investigation revealed that all nine isolates were weak biofilm formers, eight isolates showed lower SBF compared with the internal NC. SPK0043 showed almost zero SBF, while SPK0077, SPK0053 and SPK0094 produced most biofilms.

## Discussion

The epidemiology of hvKp in clinical routine diagnostics was investigated by string-testing and molecular confirmation assays. Comparisons of string-testing on common commercial solid media, under different growth conditions were realized to assess a reliable and quick method for detection of putative hvKp amongst mucoid isolates. Further, the study aimed to characterize putative hvKp isolates by WGS and phenotypic investigations.

In our prospective screening over 6 months, 100 string-test positive *K. pneumoniae* isolates from 1310 isolates were collected and tested for hypervirulence-associated genes. Nine isolates were PCR-positive, which was higher than expected compared to the previously rare reports. One of the reports on hvKp infections presented an outbreak of carbapenem-resistant putative hvKp in four healthcare facilities in Northern Germany in 2019^29,45^. From Barcelona, Spain, a study (2007–2013) reported 28 of 878 (3.1%) isolates carrying the *magA* and/or *rmpA* genes^46^. Maatallah et al. reported five putative hvKp carrying *rmpA* of 139 (3.6%) isolates analysed in a Stockholm hospital^47^. A study from the USA identified 18 hvKp of 462 (3.9%) isolates tested^48^. Until now, there is no data on prevalence of hvKp nor a surveillance system of hvKp established in Germany. Therefore, data is fragmentary both in Germany and in other countries, since most surveillance focuses on resistance traits. None of our putative hvKp isolates showed multidrug- or carbapenem resistance traits or genes. The prevalence of multidrug resistance in hvKp is low in Germany^23^. Regarding clinical materials, putative hvKp isolates originated mainly from respiratory material (n = 4) and blood cultures (n = 3). Also Ballén et al.observed a high prevalence of the *rmpA* gene in respiratory isolates^49^. Interestingly, within the high proportion of analysed urinary isolates (58%), only one was identified with hvKp-associated genes.

A multiplex-PCR was used to identify hypervirulence genes in clinical *K. pneumoniae* isolates. The WGS-based data was also used to detect hypervirulence-associated genes. By this, the nine putative hvKp isolates showed a heterogeneous equipment of virulence- associated genes. The gene *rmpA2* was identified least frequently, whereas *rmpA* was the only gene detected in all nine isolates. Sequencing revealed that the PCR detected two *rmpA2*, false positive, probably a result of manual investigation and the relatively similar product size. Different frequencies of these regulatory genes were also shown by other studies, indicating a more common distribution of *rmpA* in general^48,50^.

The *rmpA* and *rmpA2* genes mediate increased capsular polysaccharide production and are thus considered regulators of the mucoid phenotype, among other factors^19,31^. In contrast, Heiden et al*.* also showed that loss of the *rmpA* and *rmpA2* genes had no significant difference on hypermucoviscosity^29^. The same phenotypic assay was applied to our study to quantify the hmv phenotype. All isolates showed an hmv phenotype differing in expression. Isolates with ST23 and capsule type K1 are known for hypermucoviscosity^31^.

Interestingly, one ST90 isolate with capsule type K4 showed also a hvm phenotype, which was unknown for hvKp isolates. K4 was described generally associated with hypervirulent *K. pneumoniae*^51,52^. In general, the ability of biofilm formation was low in the hvKp isolates^53,54^. The results are in line with other studies as strong biofilm formation is rarely correlated with hvKp or the hmv phenotype^55,56^.

Based on such findings, hypervirulence should not be equated with hypermucoviscosity or classical virulence characteristics, e.g. biofilm formation, as we tried to differentiate in our study^29^. Nevertheless, the *rmpA* and *rmpA2* genes have been shown to increase the virulence of a strain, but may not be the best indicator for hypervirulence. Instead, Shankar et al*.* suggested aerobactin (*iutA*) as most frequent and most stable marker gene for putative hvKp isolates; our investigation also detected aerobactin in 8/9 isolates^25^. Interestingly, the one isolate without aerobactin showed also the least hypermucoviscosity in the sedimentation assay.

Sequencing data investigations further enabled molecular typing, especially assigning 7/9 isolates to ST23. This lineage is well known for its association with hypervirulence traits worldwide^57^. Further, the acquisition of the ICE*Kp* seems to be a key genomic feature for ST23 isolates^57^. The identified hypervirulence plasmid pKpVP-1 is also described a common trait for hvKp isolates^58^. The seven ST23 isolates did not belong to an outbreak or transmission event, since they were obtained from two hospitals and five different wards; further, 5/7 ST23 were isolated at patient admission. In the presented study, besides ST23 also one ST86 isolate was identified. This lineage is also known for hvKp strains and the association with pKpVP-1, but here we identified a ST86 strain with the rare plasmid variant pKpVP-2^58,59^. Unfortunately, it was not possible to fully reconstruct this plasmid from Illumina data only. The regular lack of aerobactin in pKpVP-2 might be a reason for its rare mentioning with hvKp in literature, since aerobactin seems to play an important role in hepatic infections^60^. This envisions the quest for reliable hvKp biomarkers and characteristics again, since the isolate inherit characteristics of hvKp, but is not stereotypical.

The phylogenetic investigation with other isolates from Germany led to few matching results, keeping in mind that ST23 is an international hvKp clone, but was in this database barely present. In Germany, other hvKp-associated lineages might be more common, but this example shows the urgent need for future surveillance.

The results of the string-length measurements were perused for correlations with hvKp-gene carriage for the different cultivations. Here, it was shown that the median string-lengths of the putative hvKp isolates were significantly longer compared to PCR-negative isolates for anaerobe cultivation on MH-agar plates. The significance of this hypothesis is limited due to the small sample size of the putative hvKp isolates (n = 9) and requires further investigation to confirm the correlation. A potential correlation of hypermucoviscous phenotype and hvKp pathotype has been discussed and refuted several times^14,17^. Thus, the recommendation for the identification of hvKp is to additionally use the genotype as well as the clinical manifestation of the infection^17^. Nevertheless, our results might highlight a so far unknown causal linkage. Obviously, there is a need to uniform biomarkers for detecting hvKp characteristics.

One limitation in the presented study could be the pre-selection by string-test. This might have influenced the representativeness of the collected data. However, by screening in routine diagnostics applying the string-test followed by PCR for commonly found genes (*iutA*, *rmpA*/*rmpA2*), we were able to identify otherwise undetected putative hvKp strains and to derive an estimate of the distribution of hypervirulence genes. Moreover, through subsequent sequencing we were able to detect the presence of the lineage ST23 that is internationally recognized for its association with hypervirulence, as well as to predict circulating plasmids harbouring hypervirulence genes.

## Conclusion

The associations of string-test positivity, hypermucoviscosity and hypervirulence are still difficult. We observed the, partly strong, variations of the string-test results depending on the cultivation parameters. Our results indicate a correlation between prolonged string-length with hypervirulence genes under anaerobe growth on MH-agar. We further recommend moving on from the binary definition as string-test positive or negative with the proclaimed threshold of 5 mm; and refocus on string-length measurement including cultivation parameters. These findings should further be investigated and evaluated. In summary, we endorse a prospective screening for hypervirulence traits in *K. pneumoniae*, especially isolated from respiratory materials and blood culture, in routine diagnostics.

### Supplementary Information

Supplementary Figure S1.

Supplementary Information.

## Data Availability

All data generated or analysed during this study are included in this published article and its supplementary information files. Raw sequencing data were submitted to NCBI-SRA under BioProject accession PRJNA1001661 (BioSample accession SAMN36814832–SAMN36814840).
