# Supplementary figures and images for: Detection and characterization of putative hypervirulent Klebsiella pneumoniae isolates in microbiological diagnostics
Source: Sci Rep. 2023 Nov 3;13:19025. doi: 10.1038/s41598-023-46221-w (PMC10624845; doi:10.1038/s41598-023-46221-w)

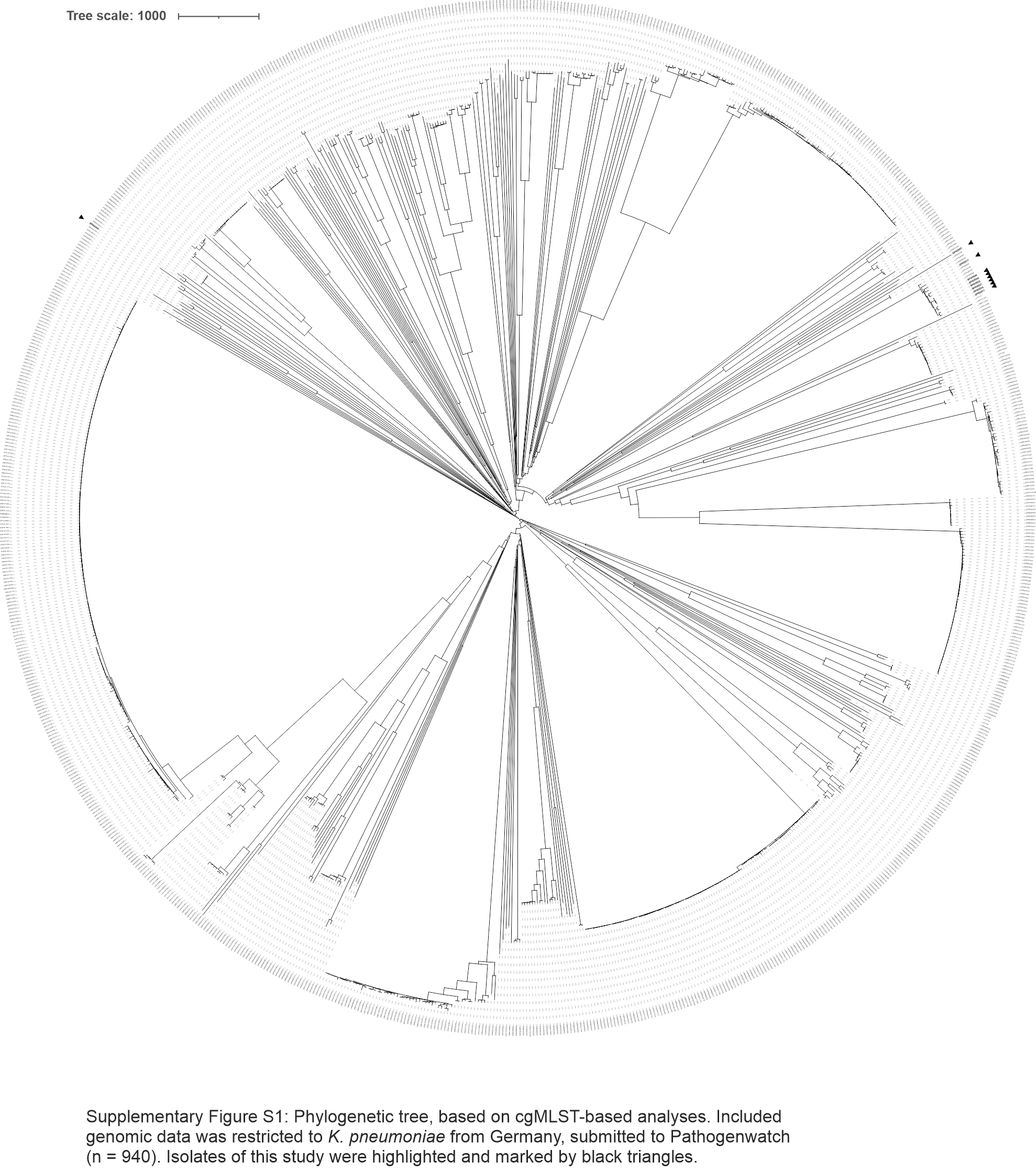

Supplement: Supplementary file 1 — Supplementary Figure S1. [file 41598_2023_46221_MOESM1_ESM.tif]
